
JOURNAL INFORMATION
==============================
NLM Title Abbreviation: ScientificWorldJournal
Journal ID: ScientificWorldJournal
NLM Journal ID: 101131163
Journal ID: TSWJ

The Scientific World Journal

ISSN: 2356-6140
EISSN: 1537-744X
Publisher: Wiley

ARTICLE INFORMATION
==============================
PMCID: PMC6084518
PMID: 30147492
DOI: 10.1100/tsw.2001.148
Article ID: 896308
Article version: 1
Subjects: Short Report

Role of Stromal Cells in Protecting Young and Aged B-Lineage Precursors from Dexamethasone-Induced Apoptosis

Pifer Jeannette M. *
Stephan Robert P.
Lill-Elghanian Deborah A.
Witte Pamela L.
Immunology and Aging Program, Loyola University Medical Center, 2160 S.1st Ave. Maywood, Illinois 60153, USA
*Jeannette M. Pifer: jpifer@lumc.edu
Publication date: 2001
Electronic publication date: 2001 Nov 10
Volume: 1
First page: 41
Last page: 41
Copyright: Copyright © 2001 Jeannette M. Pifer et al.
Copyright year: 2001
License: This is an open access article distributed under the Creative Commons Attribution License, which permits unrestricted use, distribution, and reproduction in any medium, provided the original work is properly cited.
License URL: https://creativecommons.org/licenses/by/3.0/

Funding: National Institutes of Health ROIAG13874

==============================
Miami *Nature Biotechnology* Short Reports
*TheScientificWorld* (2001) 1(S3), 41SR
ISSN 1532-2246; DOI 10.1100/tsw.2001.148

### ROLE OF STROMAL CELLS IN PROTECTING YOUNG AND AGED B-LINEAGE PRECURSORS FROM DEXAMETHASONE-INDUCED APOPTOSIS

Jeannette M. Pifer*, Robert P. Stephan, Deborah A. Lill-Elghanian, and Pamela L. Witte

Immunology and Aging Program, Loyola University Medical Center, 2160 S.1st Ave. Maywood, Illinois 60153, USA
* jpifer@lumc.edu

**INTRODUCTION.** B-lymphopoeisis is a balance between expansion and cell death. Bone marrow stromal cells support B-lymphopoeisis through direct cell-cell contact and by the production of growth factors, most notably IL-7, IGF-1, and SCF (1). Exposure to dexamethasone either *in vivo* or *in vitro* will cause the death of B-cells in early stages of development by inducing apoptosis. B-cell precursors appear to be protected from dexamethasone-induced apoptosis when co-cultured with the stromal cell line BMS2 (2). IL-7 is a possible anti-apoptotic factor since an increase in percentages of apoptotic B-cell precursors is seen in mice deficient in IL-7 production (3). However, even in IL-7 knockout mice, significant numbers of precursors still survive. Based on these findings, we hypothesized that IL-7 is the stromal derived mediator that protects B-cell precursors from dexamethasone-induced apoptosis and the protective effects of IL-7 may be augmented by the growth factors IGF-1 and SCF. Furthermore, while IL-7 has been shown to induce proliferation in primary cultures of B-lymphocytes from young animals, this effect is not seen in primary cultures of B-lymphocytes from aged animals. It is possible that, in aged animals, IL-7 acts to protect B-lymphocytes from apoptosis in a manner similar to that observed with cells in young animals.

**METHODS**. B-lymphocytes, approximately 70% pro-B cells, were harvested from Whitlock-type long-term bone marrow cultures from young (3mos) or old (22mos) BALB/c mice. Stromal cell lines were grown to 70% confluency before co-culture with pro-B cells. Cells were aliquotted into 24-well plates in 1ml volumes of $2.5 \times 10^5$ or $1 \times 10^6$ cells/ml. Dexamethasone was used at $10^{-6}$M. IL-7 (1.0 ng/ml), IGF-1 and SCF (50.0 ng/ml) were added alone and in combination to samples that were incubated at 37C for the indicated time points. Cells were harvested from wells, washed for staining with Annexin V-FITC, or washed and fixed for staining with Propidium Iodide. Samples were analyzed by flow cytometry on a FACSCalibur using 488-nm excitation (575-nm emission) for PI and 488-nm excitation (525-nm emission) for AV-FITC. IL-7 production by stromal cells was measured by bioassay.

**RESULTS.** Dex-induced apoptosis of B-cell precursors decreased in cultures containing the stromal cell lines tested regardless of levels of IL-7 production. rIL-7 and other factors produced by stromal cells were tested. Samples treated with IL-7 exhibited lower percentages of apoptosis than cells receiving no IL-7. SCF had very little effect in preventing apoptosis when administered alone or in combination with IL-7. IGF-1 alone offered protection to a lesser extent than IL-7 and the effects of these growth factors in combination was additive rather than synergistic. B-cell precursors from aged animals exhibited a slower rate of death than cells from young animals when treated with dexamethasone. Even though IL-7 failed to induce a proliferative response in cells from aged animals, preliminary evidence suggests that IL-7 may act to protect these cells from apoptosis.

**DISCUSSION.** B-lymphocytes from young and aged animals are protected from dexamethasone-induced death when cultured with stromal cells or stromal derived products. While IL-7 provides a high degree of protection, it is not the only factor capable of protecting B-lymphocytes from cell death. It is likely that IL-7 may act to influence the expression of either pro- or anti-apoptotic mediators such as bax or bcl-2 in a manner that correlates with protection against cell death.

**ACKNOWLEDGEMENT.** This work was supported by NIH Grant ROIAG13874 (to P.L.W.).

**REFERENCES.**

1. Gibson, L.F., Piktel, D., Narayann, R., Nunez, G., and Landreth, K.S. (1996) Exp. Hematol. 24, 628-637
2. Borghesi, L.A., Smithson, G., and Kincade, P.W. (1997) J. Immunol. 159, 4171-4179
3. Lu, L., Chaudhury, P., and Osmond, D.G. (1999) J. Immunol. 162, 1931-1940
